# 4D flow CMR can detect subtle right ventricular dysfunction in primary left ventricular disease

**DOI:** 10.1186/1532-429X-17-S1-Q4

**Published:** 2015-02-03

**Authors:** Alexandru G Fredriksson, Emil Svalbring, Jonatan Eriksson, Petter Dyverfeldt, Urban Alehagen, Jan E Engvall, Tino Ebbers, Carl Johan Carlhall

**Affiliations:** 1 Division of Cardiovascular Medicine, Department of Medical and Health Sciences, Linköping University, Linköping, Sweden; 2 Research and Development Unit, Örebro University Hospital, Örebro, Sweden; 3 Center for Medical Image Science and Visualization (CMIV), Linköping University, Linköping, Sweden; 4 Department of Cardiology, Institution of Medicine and Health Sciences, Linköping, Sweden; 5 Department of Clinical Physiology, Institution of Medicine and Health Sciences, Linköping, Sweden

## Background

Right ventricular (RV) function holds important prognostic value in both right- and left-sided acquired and congenital heart diseases. However, assessment of RV function remains challenging due to its complex chamber geometry and physiology. 4D flow CMR permits assessment of multidimensional intraventricular flow. We hypothesized that 4D flow specific markers of RV function can detect subtle impairment of RV function in primary left ventricular (LV) disease.

## Methods

**Figure 1:**
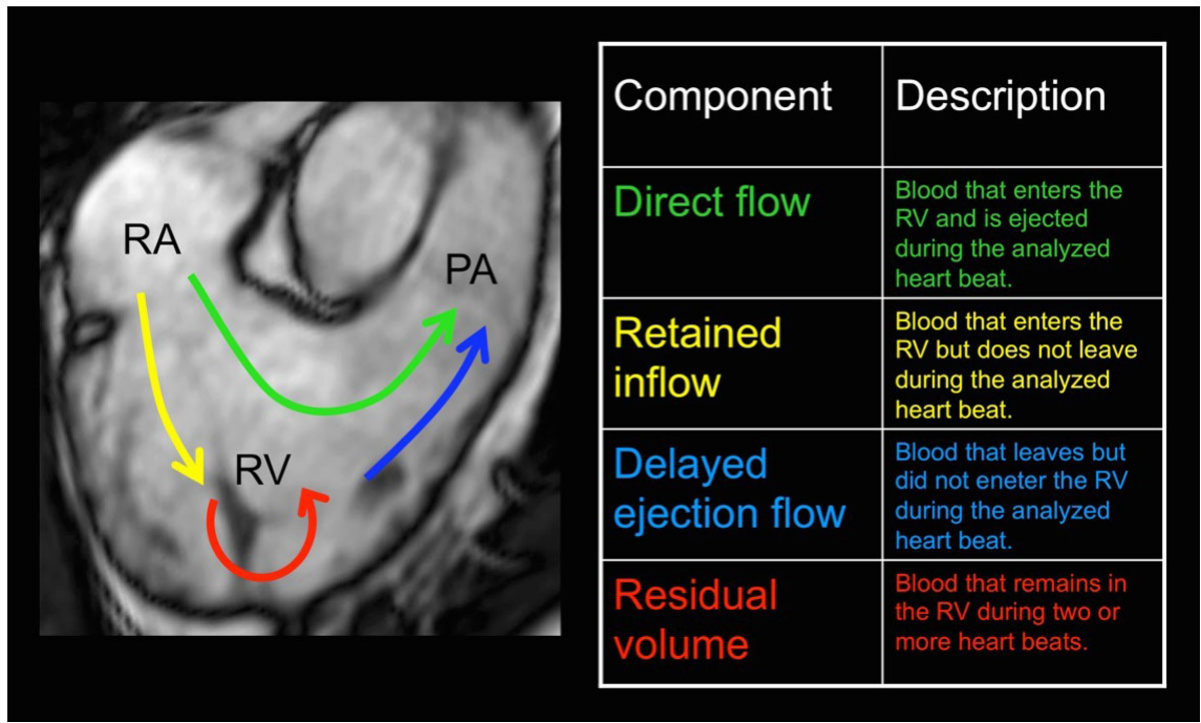
Schematic depiction of intraventricular flow components based on flow paths through the RV, here illustrated in a long axis view. RA, right atrium; RV, right ventricle; PA, pulmonary artery.

Eighteen patients with mild ischemic heart disease and eleven healthy controls were enrolled. 4D flow CMR and standard morphological images were acquired on a 3T-scanner (Philips Ingenia). The patients were stratified into two groups (n=9 each) based on LV end-diastolic volume index (EDVI) from CMR: lower LVEDVI and higher LVEDVI, respectively. The RV volume was segmented from morphological images at end-diastole (ED) and end-systole (ES) and matched to the velocity data. Pathlines were emitted from the ED volume and traced forwards and backwards in time, to the time of ES, encompassing the cardiac cycle. The blood volume was separated into functional flow components (Figure 1). The Direct Flow (DF) component was defined as RV inflow passing directly to outflow. The kinetic energy (KE) of the blood volume of the DF component was calculated throughout diastole. Echocardiographic data were also acquired and conventional RV-specific indices were analyzed for comparison.

## Results

Results are displayed in Table 1. The higher LVEDVI group had larger LVEDVI (P<0.01) and lower LV ejection fraction (P<0.01), respectively, compared to the healthy and lower LVEDI groups. No difference was found in RVEDVI and RV ejection fraction between the groups. The 4D flow specific measures "DF/EDV volume-ratio" and "DF/EDV KE-ratio at end-diastole" were lower (P<0.05 and P<0.001, respectively) in the higher LVEDVI group compared to the other two groups. There was no difference between the groups in any conventional echo Doppler indices besides the É/Á ratio which was lower (P<0.05) in the high-LVEDVI group compared to the healthy control group.

**Table 1:** Demographic and cardiac imaging data presented as mean ± SD.

Variable	Healthy controls (n = 11)	Lower LVEDVI (n = 9)	Higher LVEDVI (n = 9)
Age (years)	66 ± 4*	72 ± 4	70 ± 5

Heart rate (bpm)	67 ± 9	67 ± 11	67 ± 10

MRI derived variables			

LVEDVI (ml/m2)	67 ± 11††	63 ± 11†††	101 ± 35

LV ejection fraction (%)	64 ± 7††	68 ± 5†††	47 ± 14

MRI derived RV variables			

RV EDVI (ml/m2)	64 ± 11	59 ± 8	65 ± 14

RV Ejection fraction (%)	60 ± 7	56 ± 10	54 ± 6

DF/EDV volume ratio (%)	44 ± 6†	45 ± 6†	38 ± 5

DF/EDV KE ratio at ED (%)	65 ± 7†††	64 ± 8†††	52 ± 6

Echo derived RV variables			

RV basal diameter (mm)	32 ± 4	35 ± 7	36 ± 4

TAPSE (mm)	23 ± 4	23 ± 4	22 ± 5

RV FAC (%)	44 ± 7	47 ± 7	47 ± 6

É/Á ratio	0.76 ± 0.22†	0.60 ± 0.20	0.52 ± 0.19

RA area (cm2)	14 ± 3	16 ± 5	16 ± 3

*P<0.05 vs lower LVEDVI.

†P<0.05, ††P<0.01, †††P<0.001 vs higher LVEDVI.

LV, left ventricle; RV, right ventricle; EDVI, end-diastolic volume index; DF, Direct flow; EDV, end diastolic volume; KE, kinetic energy; ED, end-diastole; TAPSE, tricuspid annular plane systolic excursion; FAC, fractional area change; É/Á ratio, tissue velocity data in the RV free wall from early (É) and late diastole (Á), respectively; RA, right atrium.

## Conclusions

These findings suggest that in primary LV disease, mild impairment of RV function can be detected by 4D flow CMR, but not by the majority of conventional CMR and echocardiographic indices. These flow-specific alterations propose novel pathophysiological aspects of interventricular function that could add to the assessment of integrated cardiac function in the context of medical, pacing and surgical strategies.

## Funding

The Swedish Heart-Lung Foundation.

